# Impact of femoral derotation osteotomy on gait in ambulatory children with cerebral palsy: A systematic review and meta-analysis

**DOI:** 10.1016/j.bjpt.2025.101257

**Published:** 2025-10-03

**Authors:** Orsolya Z Gresits, Mátyás Vezér, Marie A Engh, Bence Szabó, Zsolt Molnár, Péter Hegyi, Tamás Terebessy

**Affiliations:** a Centre for Translational Medicine, Semmelweis University, Budapest, Hungary; b Department of Orthopaedics, Semmelweis University, Üllői út 78/b, Budapest, H-1082, Hungary; c Department of Anesthesiology and Intensive Therapy, Semmelweis University, Budapest, Hungary; d Department of Anesthesiology and Intensive Therapy, Poznan University for Medical Sciences, Poznan, Poland; e Institute of Pancreatic Diseases, Semmelweis University, Budapest, Hungary; f Institute of Translational Medicine, Medical School, University of Pécs, Pécs, Hungary

**Keywords:** Cerebral palsy, Femoral deroation osteotomy, Gait analysis

## Abstract

**Background:**

Femoral derotation osteotomies (FDRO) are commonly performed in children with cerebral palsy who present with intoeing gait. However, the impact of FDRO on gait function and long-term results remains unclear.

**Objective:**

This study aimed to quantify and qualify gait changes following gait-improving surgeries involving FDRO in ambulatory children with cerebral palsy and no associated hip pathologies, to support individualized decision-making regarding this invasive procedure.

**Methods:**

A systematic search was conducted in May 2023 across six databases. Kinetic, kinematic, temporospatial parameters, and gait scores were extracted pre- and post-FDRO. A random-effects meta-analysis was performed.

**Results:**

Forty-six articles, including 1144 patients, were analyzed. Significant improvements were observed in pelvic rotation (mean change: 6.6°, 95 % confidence interval [CI]: 2.2 to 11), hip rotation (mean change:14.4°, 95 % CI:16.7 to –12.1), foot progression angle (mean change:16.1°, 95 % CI:18.3 to –14), and gait scores (standardized mean difference [SMD]: 0.99, 95 % CI: 0.52 to 1.47). The estimated improvement in gait scores corresponded to a 10-point increase in the Gait Deviation Index in the short term and 6.9 points in the long term. No deterioration was found in any assessed parameter. Intoeing gait was corrected in 74 % of patients at one year and in 69 % at five years postoperatively. The recurrence rate was 13 %.

**Conclusion:**

While evidence suggests that femoral derotation osteotomies in ambulatory children with cerebral palsy without hip pathology improve overall gait function, the quality of available data is low. Patient-reported outcomes, including quality of life and satisfaction, are lacking. Definitive surgical indication could not be established; factors to consider include femoral anteversion, hip rotation at gait analysis, patient age, and relevant functional impairments.

## Highlights

• Femoral Derotation surgery is often advised to treat in-toeing in cerebral palsy.
• Overall results demonstrate improved gait function.
• A clear, uniform surgical indication could not be determined.
• Including the physical therapist in charge in the decision can be advised.

## Introduction

Cerebral palsy (CP) is the most common cause of physical disability in childhood, affecting 0.1–0.3 % of children.^1^ >60 % of ambulatory children with CP exhibit an intoeing gait,^2^^,^^3^ in which an increased internal hip rotation is usually a main contributor.^4^, ^5^, ^6^ Contributing factors to increased internal hip rotation include spasticity, abnormal muscle tone, contractures, hip rotator muscle imbalance, and increased femoral anteversion.^5^ Beyond aesthetic concerns, in-toeing is frequently associated with functional problems such as tripping over one's feet and knee rubbing.^7^ In many cases, it does not resolve spontaneously, and surgical correction is often recommended.^2^^,^^8^^,^^9^

The gold-standard treatment is femoral derotation osteotomy (FDRO), often performed as part of a single-event multilevel surgery to correct lever arms and improve gait.^10^ Short-term effects have been widely reported, showing a significant improvement in hip rotation and foot progression angle, and pelvic rotation in children with unilateral involvement, though not in those with bilateral involvement.^5^ Restoring lever arms may also prevent secondary deformities, potentially preserving function in the long term. These potential benefits often favor surgical intervention. However, uncertainties remain regarding the precise indications and results of FDROs.^11^ While rotation improvements are generally maintained in the long term, data on other kinematic changes and kinetic outcomes remain limited. Additionally, the possibility of recurrence^12^ raises concerns about optimal surgical timing, as recurrence rates might be higher if surgery was performed before the age 10.^5^^,^^13^

FDROs are invasive procedures with associated risks, including the need for general anesthesia, surgical complications (e.g., bleeding, non-union, under- or over-correction, fixation failure) ,^14^ and an extended rehabilitation period.^15^

FDROs in this indication are considered a treatment of choice,^7^ particularly for patients with good baseline gait function. The considerable individual variability in CP may make it even more challenging to decide whether or not to operate. This study, therefore, aims to systematically review and synthesize the results of orthopedic surgeries with FRDOs in ambulatory children with CP, where the surgery aimed to improve gait function. The main goal was to aid individual decisions about whether to undergo this invasive procedure, aiming to' optimize potential outcomes and minimize risk’.^16^

## Materials and methods

This systematic review and meta-analysis was conducted in accordance with the PRISMA 2020 guideline^17^ and the recommendations of the Cochrane Handbook for systematic reviews of interventions.^18^ The study protocol was registered on PROSPERO (CRD42022312486), and all procedures strictly adhered to it.

### Search strategy

A comprehensive search was conducted in May 2023 across the following databases: CINAHL, Cochrane CENTRAL, Embase, PubMed, Scopus, and Web of Science databases were searched in May 2023. The search terms used were ”Cerebral Palsy” AND osteotomy. For the Scopus database, the search was limited to title, abstract, and keyword fields. No other filters were applied elsewhere. The reference lists of all included studies were also manually screened for additional records.

### Eligibility criteria

Eligible studies assessed outcomes of instrumented three-dimensional gait analysis in pediatric patients with cerebral palsy (under 18 at the time of surgery) before and after undergoing a femoral derotation osteotomy (FDRO). Patients with hip conditions were excluded to prevent any distortion of femoral osteotomies performed to treat hip subluxation or luxation. Further inclusion criteria are detailed in the Supplementary Material.

### Study selection

Two reviewers independently (OG and MV) screened the titles and abstracts, followed by full text assessments of potentially eligible studies. Duplicates were removed prior to screening. Disagreements during selection were resolved through discussion, and inter-rater agreement was assessed using Cohen’s kappa coefficient.

### Data extraction

A standardized data extraction form was developed. One reviewer (OG) performed the initial data extraction, and a second reviewer (MV) independently verified the extracted data. When multiple studies utilized data from the same gait laboratory database and had overlapping timeframes, they were considered dependent. In such cases, the study with the largest sample size was retained for meta-analysis. Full details are provided in the Supplementary Material.

The following outcome measures were extracted: kinematic and kinetic parameters of the lower limbs, temporospatial variables, and composite gait scores derived from instrumented gait analysis.

### Subgroup definitions

Subgroups were defined according to time since surgery: short-term (≤2 years), mid-term (3–4 years), and long-term (≥5 years); type of cerebral palsy involvement: unilateral or bilateral; and osteotomy location on the femur: proximal vs. distal.

### Data synthesis and statistical analysis

Given the anticipated clinical heterogeneity, a random-effects model was used for all meta-analyses. Effect sizes were calculated as proportions (with 95 % confidence intervals [CIs]) for correction and recurrence rates, and as mean differences (MD) or standardized mean differences (SMD) with 95 % CIs for continuous outcomes. Proportions were computed by extracting the number of events and total participants in each study. Pooled proportions were estimated using a random intercept logistic regression model,^19^^,^^20^ and heterogeneity variance (τ²) was estimated via the maximum likelihood method. The Clopper-Pearson method^21^ was used to compute 95 % CIs for individual study proportions.

Heterogeneity was quantified using Higgins and Thompson’s I² statistic,^22^ with thresholds of 25 %, 50 %, and 75 % representing low, moderate, and high heterogeneity, respectively. Publication bias was evaluated via funnel plots and Egger’s test for small-study effects,^23^ with a p-value <0.10 considered indicative of potential bias.

All statistical analyses were made with R ^24^ using the meta ^25^ package for meta-analysis calculations and plots.

Continuous outcomes were synthesized using MDs or SMDs,^26^ depending on the scale of measurement. For these analyses, sample size, mean, and standard deviation (SD) were extracted for both baseline and follow-up measurements. If the change-from-baseline mean and SD were not reported, these were imputed using correlation coefficients derived from similar studies, as recommended by the Cochrane Handbook.^18^ Pooled MDs and SMDs were calculated using inverse-variance weighting. The heterogeneity variance (τ²) was estimated using the restricted maximum-likelihood (REML) method, and 95 % CIs were obtained using the Q-profile method.^27^^,^^28^

To facilitate clinical interpretation, SMDs were re-expressed on the original measurement scales by multiplying the pooled SMD by the pooled SD of each scale.^29^ Further details are provided in the Supplementary Material.

### Presentation of results

Results of the meta-analyses were presented using forest plots. Where sufficient data allowed and heterogeneity was acceptable, prediction intervals were also provided to estimate the likely range of effects in future studies.

### Quality assessment

Methodological quality of the included studies was assessed using the Methodological Index for Non-Randomized Studies (MINORS) tool.^30^ Controlled studies were considered high quality if they scored ≥17 out of a possible 24 points, and non-controlled studies were considered high quality with scores ≥12 out of 16. Studies scoring below these thresholds were deemed low quality, as adopted in previous systematic reviews.^31^^,^^32^

## Results

### Search and study selection

A total of 1427 records were screened, from which 75 full-text articles were retrieved for further evaluation. Ultimately, 46 articles from 26 independent studies or databases met the eligibility criteria and were included in the final analysis.^12^^,^^13^^,^^33^, ^34^, ^35^, ^36^, ^37^, ^38^, ^39^, ^40^, ^41^, ^42^, ^43^, ^44^, ^45^, ^46^, ^47^, ^48^, ^49^, ^50^, ^51^, ^52^, ^53^, ^54^, ^55^, ^56^, ^57^, ^58^, ^59^, ^60^, ^61^, ^62^, ^63^, ^64^, ^65^, ^66^, ^67^, ^68^, ^69^, ^70^, ^71^, ^72^, ^73^, ^74^, ^75^, ^76^ The study selection process is illustrated in the PRISMA flow diagram (Figure supplementary 1). Baseline characteristics of the included studies are presented in Appendix A1. In total, 1144 patients were assessed. Only one study^43^ reported outcomes following isolated femoral derotation osteotomies (FDROs), while all others combined FDROs with soft tissue procedures, additional bony corrections, or both.

### Methodological quality and risk of bias

Methodological quality, assessed using the MINORS tool, and publication bias assessments via funnel plots are presented in the Supplementary Material. Only six studies were classified as high quality.^35^, ^36^, ^37^, ^38^, ^39^, ^40^, ^41^, ^42^, ^43^, ^44^, ^45^, ^46^, ^47^, ^48^, ^49^, ^50^, ^51^, ^52^, ^53^, ^54^, ^55^, ^56^, ^57^, ^58^, ^59^, ^60^, ^61^, ^62^, ^63^, ^64^, ^65^, ^66^, ^67^, ^68^, ^69^, ^70^, ^71^, ^72^ A sensitivity analysis was performed to determine whether data from high- and low-quality studies produced divergent results. For hip rotation (Figure supplementary 2) and foot progression angle (Figure supplementary 3), no significant differences were observed; therefore, all studies were retained in the meta-analysis. Twelve studies had prospective designs.^37^, ^38^, ^39^^,^^43^^,^^47^^,^^48^^,^^63^^,^^64^^,^^66^^,^^68^^,^^72^^,^^75^ Funnel plots indicated a low likelihood of publication bias.

### Results of meta-analyses

**Fig. 1:**
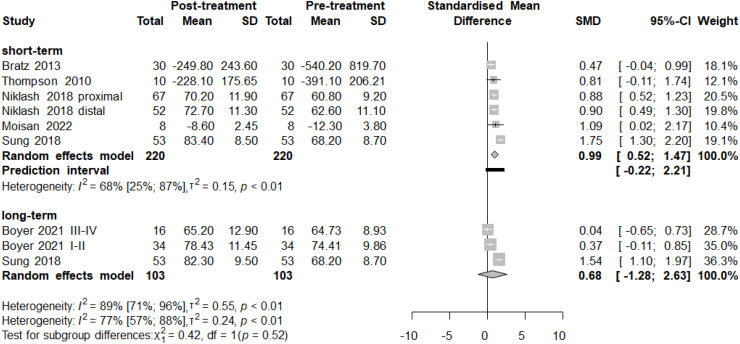
Forest plot of pooled gait score results. Gait scores are single scores representing the quality of patient kinematics during gait. As three different scores (GDI, GPS, and GGI) were used among the articles, standardized mean differences (SMD) were calculated. In the short-term analysis (220 patients), a significant improvement was revealed after FDRO. The long-term analysis (103 patients) also reveals a tendency for improvement, but the results are not significant. Overall heterogeneity (I2 value of 77 %) is high, presumably because of the large individual differences observed in Cerebral Palsy). Prediction intervals (i.e., the expected range of effects of future studies) suggest, that future studies will likely have similar results.

**Fig. 2:**
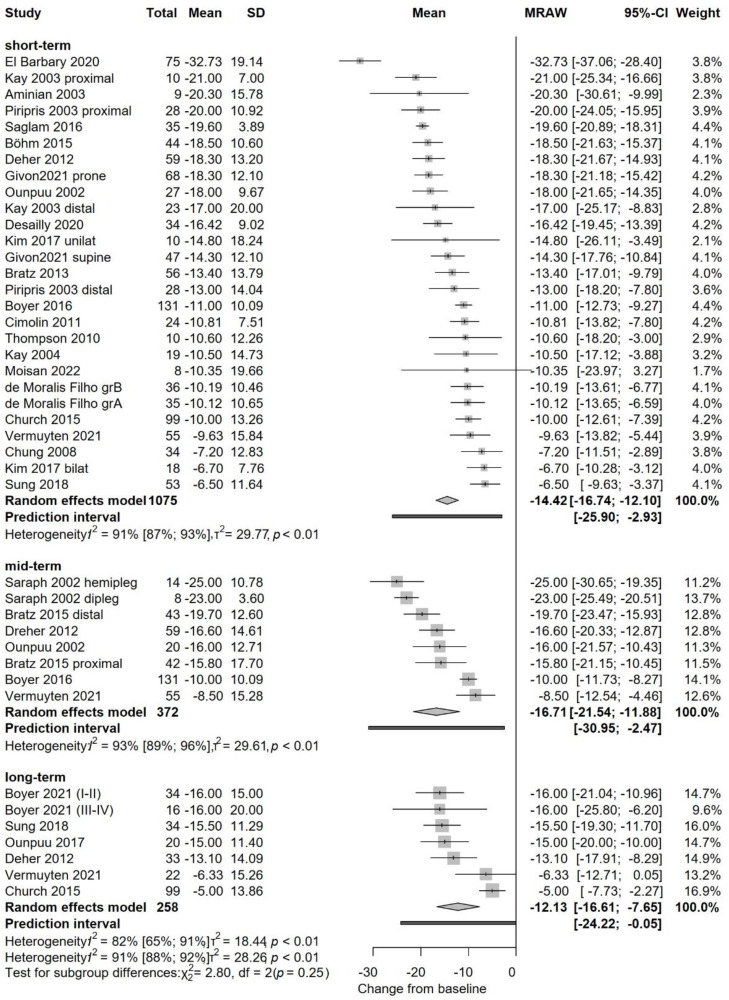
Forest plot of pooled hip rotation results. Results are presented in degrees, negative values represent internal rotation. All included studies reported improvements, magnitude was between −32 and −5 degrees. Short-term analysis (1–2 years after the operation; 1075 patients) revealed a significant improvement in internal hip rotation with a mean change of −14.4 degrees. Mid-term analysis (3–4 years after the operation; 372 patients) revealed a similar, significant improvement of 16.7 degrees. Long-term analysis (>5 years after the operation, 258 patients) had also a significant improvement of 12.1 degrees. Heterogeneity was high in all analyses. Prediction intervals (i.e., the expected range of effects of future studies) suggest, that future studies will likely have similar results.

**Fig. 3:**
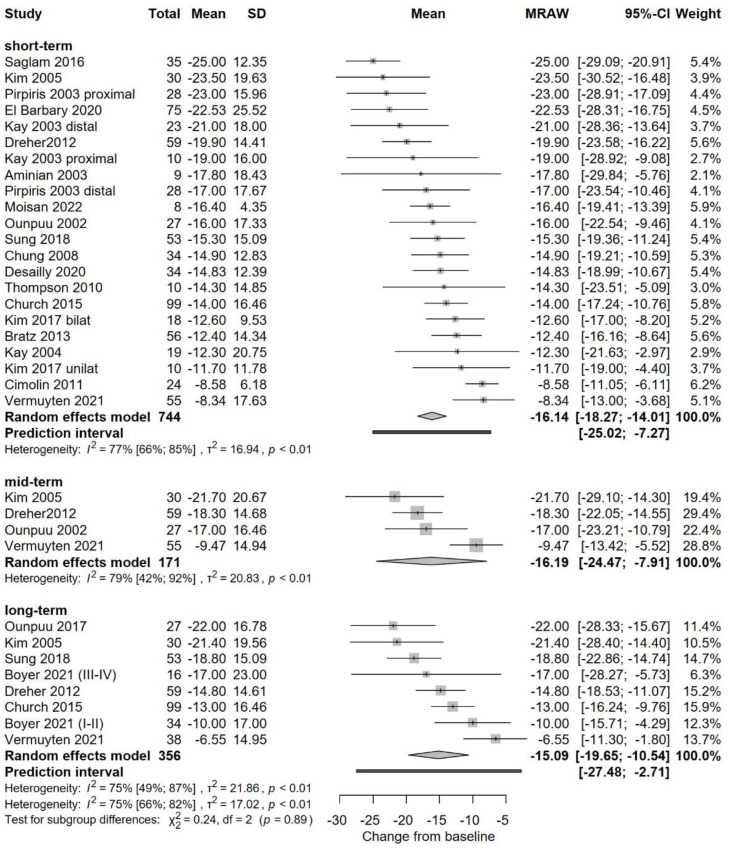
Forest plot of pooled foot progression angle results. Results are presented in degrees, negative values represent internal rotation. All included studies reported improvements, magnitude was between −25 and −6.5 degrees. Short-term analysis (1–2 years after the operation; 744 patients) revealed a significant improvement with a mean change of −16.1 degrees. Mid-term analysis (3–4 years after the operation; 171 patients) revealed a similar, significant improvement of 16.1 degrees. Long-term analysis (>5 years after the operation, 356 patients) also showed a significant improvement of 15.1 degrees. Heterogeneity was high in all analyses. Prediction intervals (i.e., the expected range of effects of future studies) suggest, that future studies will likely have similar results.

A summary of the pooled statistical results is presented in Table 1. Outcomes with sufficient data for meta-analysis included: overall gait scores (Fig 1), Gait Deviation Index (GDI, Figure supplementary 4), Gait Profile Score (GPS, Figure supplementary 5), Gillette Gait Index (GGI, Figure supplementary 6), pelvic rotation (Figure supplementary 7), hip rotation (Fig 2), foot progression angle (Fig 3), pelvic tilt (Figure supplementary 8), knee flexion-extension (Figure supplementary 9), hip abduction-adduction (Figure supplementary 10), cadence (Figure supplementary 11), step length (Figure supplementary 12), stride length (Figure supplementary 13), step width (Figure supplementary 14), and walking velocity (Figure supplementary 15).

**Table 1:** Summary of pooled kinematics and temporospatial statistical results. Statistically significant changes are marked with*.

Outcome, dimension	Minimal Clinically Important Difference^a^	Minimal Detectable Change	short-term (1–2 years after FDRO)	mid-term (3–4 years after FDRO)	long-term (>5 years after FDRO)	Forest plot number
gait scores^37^^,-^^72^ SMD	N.A.	N.A.	SMD 0.99 (CI 0.52 to 1.47)*	–	SMD 0.68 (CI −1.28 to 2.63)	Fig. 2
Gait Deviation Index (GDI)	5^98^	N.A.	MD 9.25 (CI 4.9 to 13.6)*	–	–	Fig.S4
Gait Profile Score (GPS) degrees	1.6^99^	N.A.	MD −6.36° (CI −12.51 to −0.21)*	–	–	Fig.S5
Gillette Gait Index (GGI)	N.A.	N.A.	MD 186.9 (CI 70 to 303)*	–	–	Fig.S6
pelvic rotation preop. asymmetric group^33^^,-^^72^ degrees	4.1^100^	N.A.	MD 6.64° (CI 2.21 to 11.07)*	–	–	Fig.S7
pelvic rotation preop. symmetric group^12^^,–^^74^ degrees	4.^97^	N.A.	MD 1.05° (CI 0.42 to 1.68)*	–	–	Fig.S7
hip rotation^12^^,^^33^, ^34^, ^35^^,^^37^, ^38^, ^39^^,^^41^, ^42^, ^43^^,^^45^^,^^46^^,^^48^^,^^49^^,^^51^, ^52^, ^53^^,^^57^^,^^63^^,^^64^^,^^66^, ^67^, ^68^^,^^70^^,^^72^^,^^74^ degrees	7.9^97^	N.A.	MD −14.42° (CI −16.74 to −12.10)*	MD −16.71° (CI −21.54 to −11.88)*	MD −12.13° (CI −16.61 to −7.65)*	Fig. 3
foot progression angle^12^^,^^13^^,^^33^, ^34^, ^35^^,^^37^, ^38^, ^39^^,^^41^, ^42^, ^43^^,^^45^^,^^46^^,^^48^^,^^49^^,^^51^, ^52^, ^53^^,^^57^^,^^63^^,^^64^^,^^66^, ^67^, ^68^^,^^70^^,^^72^^,^^74^ degrees	N.A.	N.A.	MD −16.14° (CI −18.27 to −14.01)*	MD −16.19° (CI −24.47 to −7.91)*	MD −15.09° (CI −19.65 to −10.54)*	Fig. 4
pelvic tilt^49^^,–^^74^ degrees	4.8^97^	N.A.	MD −1.39° (CI −2.8 to 0.02)	–	–	Fig.S8
knee flexion-extension^34^^,–^^73^ degrees	4.2^97^	N.A.	MD −8.63° (CI −13.01 to −4.24)*	–	–	Fig.S9
hip ab-adduction^49^^,–^^101^ degrees	N.A.	N.A.	MD −4.32° varisation (CI −11.36 to 2.72)	–	–	Fig.S10
cadence^48^^,–^^67^ steps/min	8.1 % of normal^99^	5 ^102^	MD −1.51 (CI −21.2 to 54.93)	–	–	Fig.S11
step length^42^^,–^^66^ centimeters	N.A.	0.83^103^	MD −0.37 (CI −4.34 to 3.6)	–	–	Fig.S12
stride length^48^^,–^^67^ centimeters	5.8 % of normal^99^	3.6 ^102^	MD −0.68 (CI −7.83 to 6.46)	–	–	Fig.S13
step width^42^^,–^^66^ centimeters	N.A.	0.95^99^	MD −2.55 (CI −3.78 to 1.33)	–	–	Fig.S14
*velocity**^33^**^,–^*^67^ m*/sec*	9.1 % of normal^99^	5.6 ^102^	MD 0.05 (CI −0.18 to 0.27)	MD 0.09 (CI −0.13 to 1.04)	–	Fig.S15

Abbr. CI – confidence interval, MD – mean difference, SMD – standardized mean difference.

a If Minimal Clinically Important Changes were not available for children with cerebral palsy, reported clinically relevant amounts of changes for the same population were used. If not available, for typically developing children or adults with CP.

Gait scores^77^^,^^78^ are composite indicators of gait quality. The GDI,^79^ GPS,^80^ and GGI^81^ were utilized, each with distinct scales and calculation methods (detailed in the Supplementary Material). Results of different gait scores are not directly comparable. Pooled short-term results from six independent studies^37^, ^38^, ^39^, ^40^, ^41^, ^42^, ^43^, ^44^, ^45^, ^46^, ^47^, ^48^, ^49^, ^50^, ^51^, ^52^, ^53^, ^54^, ^55^, ^56^, ^57^, ^58^, ^59^, ^60^, ^61^, ^62^, ^63^, ^64^, ^65^, ^66^, ^67^, ^68^, ^69^, ^70^, ^71^, ^72^ demonstrated significant improvement following FDRO, with a standardized mean difference (SMD) of 0.99 (95 % CI: 0.52 to 1.47). Retransformed values were approximately +10.1 points on the GDI, –1.6 points on the GPS, and –394.1 points on the GGI. In the long-term, the pooled SMD was 0.68 (95 % CI:1.28 to 2.63), corresponding to approximately +6.9 on the GDI, –1.0 on the GPS, and –270.7 on the GGI.

### Kinetic effects of FDRO

Boyer et al.^37^ reported a minimal reduction in hip abduction moments ten years post-surgery. Niklasch et al.^60^ identified that patients with recurrent internal rotation gait had significantly lower preoperative hip joint impulse. Thielen et al.^71^ observed increased frontal plane hip moments one year after supracondylar FDRO. Sample sizes were small.

### Effect of osteotomy localization

Subgroup analysis comparing proximal versus distal FDRO sites showed no statistically significant differences in hip rotation (Figure supplementary 16) or foot progression angle (Figure supplementary 17).

### Comparative outcomes: FDRO versus no FDRO

Nine articles from seven independent studies included control groups. Of these, only three^35^, ^36^, ^37^, ^38^, ^39^, ^40^, ^41^, ^42^, ^43^, ^44^, ^45^, ^46^, ^47^, ^48^, ^49^, ^50^, ^51^, ^52^, ^53^, ^54^, ^55^, ^56^, ^57^, ^58^, ^59^, ^60^, ^61^, ^62^, ^63^, ^64^, ^65^, ^66^, ^67^, ^68^, ^69^, ^70^, ^71^, ^72^, ^73^, ^74^, ^75^ used age-matched controls with internal hip rotation gait who did not undergo FDRO (see Supplementary for full details). All studies except Kay^52^ reported superior outcomes for the FDRO groups. Pooled data showed a short-term mean difference (MD) of –10.13° (95 % CI:21.8 to 1.54) in hip rotation (Figure supplementary 18) and –7.18° (95 % CI:17.5 to 3.14) in foot progression angle (Figure supplementary 19).

### Pain

Only McMulkin et al.^56^ evaluated pain, reporting significantly reduced pain one year after FDRO in GMFCS level I/II patients. Some improvement was also observed in level III patients.

### Adverse events

Eleven of the 46 included studies reported adverse events. All were surgical complications, such as non-union requiring revision surgery. No anesthesia-related or life-threatening complications were reported. Detailed descriptions are provided in the Supplementary Material.

### Correction rate

Successful correction was defined as postoperative hip rotation within a functional range; specific thresholds varied across studies (see Supplementary). The pooled correction rate was 74 % (95 % CI: 52 % to 96 %) in the short term and 69 % (95 % CI: 52 % to 88 %) in the long term (Fig 4a). Ounpuu et al.^64^ reported a correction rate of 59 % at one year and 52 % at ten years.

**Fig. 4a:**
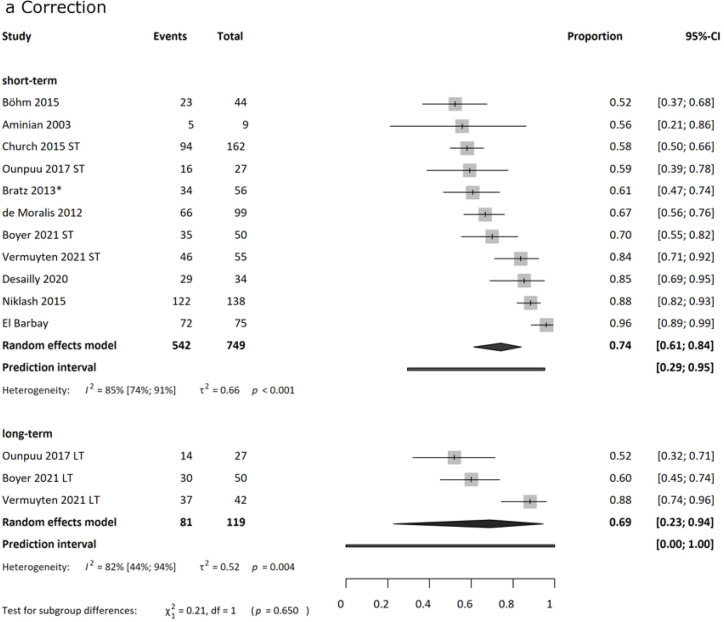
Forest plot of pooled correction rate. Results are presented as proportions. Total patient numbers and the number of patients reported to be reaching a good correction are presented The definition of correction varied across the articles; the ones with lower correction rates applied more rigorous criteria. Short-term analysis (749 patients) revealed a pooled correction rate of 74 %. Long-term analysis (119 patients) shows a rate of 69 %. Heterogeneity was high in all analyses.

### Recurrence

Recurrence of internal rotation gait after initially successful FDRO was reported in 3 % to 33 % of cases, with a mean recurrence rate of 13 % (Fig 4b).

**Fig. 4b:**
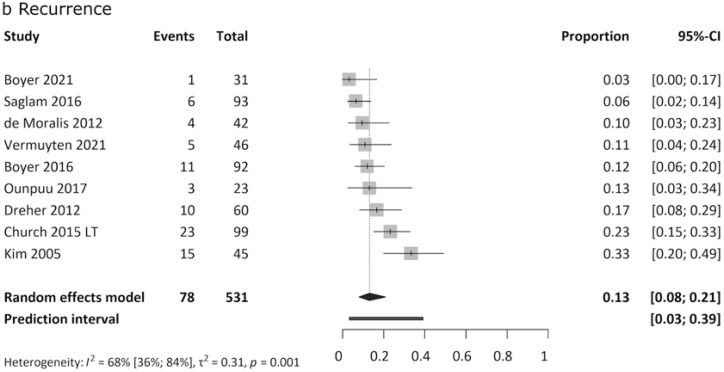
Forest plot of pooled recurrence rate. Results are presented as proportions. Total patient numbers and the number of patients showing a recurrence are presented. The first row shows the mean age of patients at the surgery in each study. Analysis of 531 patients revealed a pooled recurrence rate of 13 %. Heterogeneity was moderate.

### Quality of life and patient satisfaction

No studies included measures of quality of life or patient satisfaction.

## Discussion

This systematic review and meta-analysis synthesized evidence from 46 articles evaluating the outcomes of femoral derotation osteotomy (FDRO) in ambulatory children and adolescents with cerebral palsy without associated hip pathologies. Overall results support the general belief that FDROs improve gait function in the study population. Changes to expect are evident in pelvic rotation, hip rotation, hip abduction/adduction, and foot progression angle. Additional studies are needed to examine other kinematic changes, kinetics, and walking energy.

The most significant change is the direct impact of FDRO on transverse plane kinematics. A consistent improvement in internal hip rotation was observed, with an average of −14 degrees, twice the Minimal Clinically Important Difference (MCID). Consequently, in-toeing improved as the progression angle changed with an average of −16 degrees. Comparable magnitudes suggest that femoral anteversion was a major contributor to in-toeing. The improvements are likely significant enough to lead to a meaningful enhancement in the functional problems as well.^64^ Favorable results were maintained over more than five years. The results indicate that patients with functional issues related to internal rotation gait presumably experience benefits from surgery.

Pelvic rotation is problematic in 30–60 % of children with CP.^82^, ^83^, ^84^ Articles with symmetric baseline data revealed a pooled minor mean change of 1°, while it was 6.6° for asymmetric ones. FDRO presumably improves pelvic malrotation with 1.5 times the MCID. This finding is consistent with Hara et al.,^85^ reporting more correction following FDRO in the case of greater pre-operative pelvic asymmetry.

Pooled results confirmed a hip varisation of −4 degrees. This change is mainly the direct effect of FDRO, as it also changes the projection of the femur in the frontal plane, even if the osteotomy was purely rotational.^86^

Pelvic tilt results were too heterogeneous to draw a clear conclusion. Studies reporting improvements performed FDROs in proximal localization; the others were distal,^62^^,^^68^ mixed,^67^ or no data.^49^ This supports the assumption that proximal FDOs may amend increased anterior pelvic tilt, but distal FDOs do not.^55^, ^56^, ^57^, ^58^, ^59^, ^60^, ^61^, ^62^

We attribute the favorable change of 8.6° toward more extended knees mainly to be the consequence of simultaneously performed hamstring lengthenings. However, contrary opinions exist. Akalan et al.^87^ report that peak knee extension is influenced by femoral anteversion both in typically developing children and in CP. The lever-arm change can also have some knee-extension effects. As the foot progress angle normalizes, the center of pressure of application of the ground reaction force moves closer to normal, which lengthens the knee-extensor moment arm.^88^

In relation to kinetic results, studies were scarce, although the primary goal of orthopedic surgeries is to restore lever arms.^88^ Changing anteversion directly influences hip kinetics in anatomic modes,^86^^,^^89^ although the proximal bony geometry has not been altered.^71^ Boyer^37^ described an unexpected lack of improvement in hip abductor moment after FDRO, which appeared three years later. Further studies are required to confirm whether the theoretically favorable kinetic changes are observed in real life.

As expected, this systematic review shows that surgery did not influence temporospatial gait parameters.

Regarding the gait function, a consistent improvement was observed across all articles reporting gait scores.

Results are heterogeneous, probably because even a cleaned CP population varies significantly. The magnitude of improvement in the short term was approximately 10-point GDI, which is two times as large as the MCID and represents a change equal to 1 SD in the gait of healthy subjects.^78^ Some deterioration was observed in the long-term function. Supporting the belief that gait function in CP tends to deteriorate with growth. More severe categories either show less improvement, have a higher rate of deterioration, or both. Improvement seems to be maintained in the long term, in accordance with Saisongcroh et al.^90^ However, the only controlled study with long-term results^37^ reported that the FDRO group had a significant advantage only in the short term but not 10 years after surgery. Lennon et al.^91^ also proposed that short-term superior gait results of orthopedic surgeries might no longer be present in adulthood.

It is difficult to determine the real-life impact of gait score improvement, as there is limited data on quality of life or client satisfaction. Theoretically, it should be significant. McMulkin et al.^56^ compared multilevel surgery with and without FDROs. The FDRO group had better gait outcomes, with an average GDI improvement of 13 points and a 15 % reduction of net oxygen cost. However, they were unable to demonstrate that the improved kinematics led to lower metabolic power. Gill et al.^92^ however, concluded that GDI affected metabolic power approximately twice as much as the next most significant contributor. According to their calculations, a 13-point improvement in GDI would be equivalent to a 10–22 % reduction in metabolic power. Gagnat et al.^93^ also associated increased gait deviation to increased energy cost of walking in GMFCS I and II.

Additional studies are needed to clarify whether and to what extent improved gait function after FDROs is linked to real-life benefits, such as reduced walking energy, less fatigue, or longer walking distance.

The self-reported rate of successful FDROs was between 52 % and 96 % in this systematic review. The difference lies mainly in how success is defined. Articles with lower rates applied more rigorous criteria; hip rotation had to fall in the normal range of typically developing children. Results confirm that perfect correction was achieved in over half of the cases. The exact rate of under-correction was stated only in 2 articles, which were 4 %^48^ and 11 %.^59^

The overall recurrence rate of 13 % can be considered low, but it also had significant heterogeneity (3 % to 33 %). Identification of underlying causes is beyond this work. Reported risk factors are pre-operatively reduced hip joint impulse, increased ankle plantar flexion, internal foot progression,^60^ and younger age at surgery (<10 years) .^5^^,^^13^

In 2014, a meta-analysis was performed^5^ on FDRO; however, it has several limitations to note. It did not exclude patients with hip problems; not all patients involved had FDRO, only hip and pelvic rotation kinematics were described, and the follow-up time was also limited, with a maximum of 3.1 years.

In 2024, a new meta-analysis was published^7^ reporting only long-term (5+ years) results of hip rotation kinematics, foot progression, and hip rotation passive range of motions. Statistical results show only SMDs, so the magnitudes of changes are unknown.

In terms of the strengths of our study, we highlight that we followed our pre-registered protocol and applied a rigorous methodology. Although there are large individual differences in the patients included due to the nature of CP, the study population was as homogeneous as it could be. Similarly, the concomitant procedures performed with FDRO are heterogeneous but also represent the individual needs of involved patients. The gait data of 1144 patients were included. Most gait parameters, most importantly gait scores, demonstrated consistent improvement.

The main limitations of this systematic review are the methodological designs and the low quality of the articles included. Only three studies had adequate control groups, all with limited numbers of patients and short follow-up times. Of the 46 articles included, only six were of high quality. A large proportion of our data came from retrospective cohort analyses. These studies do not represent all operated patients – as would be desirable – only the ones who had gait analyses before and after FDRO; hence, they are subject to numerous biases, systematic errors, and missing results. The goal of this study was not fully achieved due to the low data quality, the absence of subjective impact information, and the lack of practical connection between results and clinical decision-making. Also, a remarkable heterogeneity was revealed in most analyses, which could be explained by the heterogeneous nature of CP.

Future studies are needed to clarify kinetics and walking energy changes. It is advisable to follow reporting guidelines to achieve higher quality; longer follow-up times and prospective designs are recommended. Proper maintenance and analysis of CP registers would also be beneficial to clarify whether improvements from childhood orthopedic surgeries last through adolescence or adulthood. Collecting subjective outcomes is also recommended.

The most important changes to expect after FDRO seem well-defined. However, clear and universal surgical indications could not be made. Still, some points to consider can be suggested. FDROs should presumably be avoided in patients without significant internal hip rotation in gait analysis and increased femoral anteversion, as described by Schwartz et al.^69^ Therefore, gait analysis and measurement of anteversion should always be performed before considering surgery.

Patients experiencing significant functional or aesthetic problems due to internal rotation gait may benefit from FDRO.

For younger patients, presumably, a conservative treatment approach should be favored over surgery. This allows for observation of the problem's progression (or improvement) and might help avoid a higher relapse rate associated with younger age.

Physical therapists often have frequent, hands-on interactions with the child.^94^ This ongoing relationship allows physical therapists to develop a deep understanding of the child's day-to-day challenges and progress over time. Therefore, they could aid in deciding whether to operate or not, as well as the optimal timing in case of surgery. Also, they could maximize the benefits of surgery through preoperative prehabilitation^95^ and proper postoperative rehabilitation.^96^^,^^97^ Therefore, we suggest that the patients' physical therapist should be actively involved in the individual decision about FDRO.

## Conclusion

Results of this systematic review demonstrated that femoral derotation osteotomies in ambulatory children with cerebral palsy without associated hip pathology improve overall gait function, with no evidence of deterioration. However, the quality of evidence remains low, and data on patient-reported outcomes are lacking. These findings support the functional benefits of FDRO, but individualized surgical decisions should consider femoral anteversion, hip rotation patterns, patient age, and specific functional impairments.

## Ethical approval

No ethical approval was required for this systematic review with meta-analysis, as all data were already published in peer-reviewed journals. No patients were involved in the design, conduct or interpretation of our study.

The datasets used in this study can be found in the full-text articles included in the systematic review and meta-analysis.

## CRediT authorship contribution statement

**Orsolya Z Gresits:** Conceptualization, Project administration, Methodology, Formal analysis, Writing – original draft. **Mátyás Vezér:** Conceptualization, Formal analysis, Visualization, Writing – review & editing. **Marie A Engh:** Conceptualization, Data curation, Writing – review & editing. **Bence Szabó:** Conceptualization, Data curation, Writing – review & editing. **Zsolt Molnár:** Conceptualization, Funding acquisition, Writing – review & editing. **Péter Hegyi:** Conceptualization, Writing – review & editing. **Tamás Terebessy:** Conceptualization, Supervision, Writing – original draft.

## Declaration of competing interest

The authors declare no competing interest.

## Appendices

Appendix 1 (A1) Baseline characteristics of included studies

## Appendix B Supplementary materials

Image, application 1
