# Supplementary material for: Impact of femoral derotation osteotomy on gait in ambulatory children with cerebral palsy: A systematic review and meta-analysis
Source: Braz J Phys Ther. 2025 Oct 3;30(1):101257. doi: 10.1016/j.bjpt.2025.101257 (PMC12519288; doi:10.1016/j.bjpt.2025.101257)
Supplement: Supplementary file 1 [file mmc1.docx]

**Supplementary Material**

**Table of contents:**

**A, Eligibility criteria**

**B, Selection** process

**C**, **Rules for deciding the independence of eligible studies**

**D, Gait scores**

**E, More details on data analysis**

**F, Controlled Studies**

**G, Supplementary Figures**

**H, Adverse Events**

**I, Good Correction Definition**

**J, Funnel plots**

**K, Excluded articles**

**L, References**

**A, Detailed eligibility criteria authors applied at the article selection:**

**Population**: cerebral palsy patients with the ability to walk, under 18 years at the time of surgery. Exclusion: Any other neurological conditions or diseases, and individuals not capable of the walk required for instrumented 3D gait analysis.

Rules for partially overlapping population: If separate data were available for the eligible section, only that part was used. If individual data were unavailable, the study was included if most of the participants were in the predefined population.

**Intervention:** Femoral derotation osteotomy (FDRO), performed either alone or as a part of complex surgery. Both proximal (intertrochanteric or subtrochanteric level) and distal (supracondylar level) localization are accepted. Exclusion: surgical procedures without FDRO.

**Eligible study types**: any study design with at least 10 included patients, exclusively from peer-reviewed sources. Exclusion: gray literature, case reports, and case series.
The authors included only studies where instrumented 3D gait analyses were performed both before FDRO surgery and after the recovery.

**Outcomes** of authors’ interests:
1, Gait scores: Gait scores are calculated from the results of the 3D gait analysis, for example, Gait Deviation Index and Gait Profile Score.

2, temporospatial gait parameters,

3, pelvic, hip, knee, and ankle kinetics and kinematics, foot progression angle,

4, pain,

5, quality of life,

6, patient or family satisfaction,

7, adverse event(s).

**B, The selection** process was performed by two authors separately (OG and VM). After duplicate removal, authors independently screened the first titles and abstracts according to the previously set rules (mentioned above). Full texts were obtained when an article met the inclusion criteria or when there was doubt over eligibility. The remaining full texts were screened similarly. To quantify agreement, we calculated Cohen’s kappa values. Disagreements were resolved by discussion.

References of eligible studies were also searched for potential further eligible studies.

**Data extraction and analysis:** a standardized data collection sheet was prepared. One author (OG) was responsible for data extraction; a second author (MV) reviewed them.

**C, Rules for deciding the independence of eligible studies:** Studies or databases were considered independent if they undoubtedly had different populations. Publications from the same gait laboratory database were considered not independent unless the time of the measurements ensured that the population did not overlap at all. Partially overlapping populations were considered not independent. All relevant data were extracted from every study. From the non-independent studies, the most numerous data were always used in the statistical analysis.

Eligible studies were grouped:

1. Aminian 2003;
2. Böhm 2015;
3. Boyer 2016, Boyer 2017, Boyer 2021, Schwartz 2014;
4. Braatz 2013, Braatz 2018;
5. Braatz 2015;
6. Chung 2008;
7. Church 2015, Perotti 2019;
8. Cimolin 2011;
9. De Morais 2012, 2013;
10. Desailly 2020;
11. Dreher 2007, Dreher 2012, all Niklasch articles (3 articles from 2015 and 2 from 2018), Thielen 2019,
12. El Barbary 2020;
13. Givon 2021;
14. Hayford 2021;
15. Kay 2004, Kay 2005,
16. Kim 2005, 2018; Wren 2013, Wren 2022
17. Kuo 1998;
18. Kwon 2013, Sung 2018;
19. McMulkin 2016;
20. Moisan 2022;
21. Ounpuu 2002, 2017;
22. Piriris 2003;
23. Saglam 2016;
24. Saraph 2002;
25. Thompson 2010;
26. Van Campenhout 2019, Vermuyten 2021;

**D. Data analysis:** In a ***random-effects*** meta-analysis, we assume that the true effects vary across studies. The variance of these true effects is represented by τ2. REML is one of the most commonly used methods for estimating τ2 because it reduces bias compared to the standard Maximum Likelihood (ML) approach, which makes it more suitable for cases when the number of studies is relatively low.

***Retransformation of SMDs*** to all the utilized original scales. As for the discussion of the significance of the results, only the confidence intervals for the SMDs were examined. The retransformation using estimated pooled SD-s carried the risk of introducing errors to the retransformed values. These confidence intervals, therefore, were not retransformed to the scales initially utilized, only the point estimates as reference points.

**E. Gait scores** ^1,2^ are single scores representing the quality of the patient's kinematics during gait. Their calculation methods and scales differ; therefore, distinct gait scores are not directly comparable.

**Gait Deviation Index (GDI)** ^3^ is calculated from 15 standard kinematic graphs of the pelvis, hips, knees, and ankles. Ranges 0-100 points. Higher values indicate better gait: 100 points (and above) represent a normal gait. Every 10-point decrease means one standard deviation distance from the mean of the healthy individuals.

**Gait Profile Score (GPS)** ^4^ represents the root mean square difference between the patient’s kinematic data and the average of healthy subjects measured in degrees. Healthy subjects’ GPS is around 5-6°. Higher GPS scores mean more deviation from the normal gait. The minimal clinically important difference is 1.6°. ^5^

**Gillette Gait Index (GGI)** ^6^ uses 13 kinematic values of the pelvis, hip, knee, ankle, and three temporospatial parameters (percentage of stance phase, normalized velocity, and cadence). Lower scores represent better gait. An average GGI score is reported to be 15 for healthy subjects and 900 for the affected side of Type IV hemiplegics. ^7^ The square root of GGI correlates well with GPS.

**F. Controlled studies:**

| Article | No.  FDRO | No. control | Control description | Follow-up | Results | Comments |
| --- | --- | --- | --- | --- | --- | --- |
| Boyer 2016 | 86 | 108 | age-matched CP patients with increased anteversion without FDRO | ’short-term’ and long-term’ | An FDRO is an effective way to correct anteversion in children with CP. In the long term, however, FDRO is not superior to a reasonably well-matched non-FDRO control group. Rates of problematic recurrence and remodeling are low and do not differ between the groups. | Retrospective cohort analysis, the control group had less anteversion at baseline. |
| Boyer 2021 | 50 | 11 | age-matched CP patients with increased anteversion without FDRO | 10+ years (patients were at least 25 years old) | In the control group, both the anteversion and the internal hip rotation decreased to a small extent over the 10-year follow-up time. FDRO patients had significantly better results 5 years postoperatively, the difference still existed but was no longer significant 10 years postoperatively. | íParticipants of earlier studies were called back for a long-term assessment, approx. 30% showed up. |
| McMulkin 2016 | 67 | 67 | children with CP who had an indication for FDRO, but the osteotomy was not performed. | 1 year | Overall gait kinematic improvements were significantly greater when an FDRO was included in the surgical management. | - |
| Wren 2013 | 7 limbs | 32 limbs | children with CP who had an indication for FDRO, but the osteotomy was not performed | 1 year | Gait analysis can improve outcomes when its recommendations are incorporated in the treatment plan. | - |
| Chung 2008 | 34 | 18 | Single-event multilevel surgery with or without FDRO included | 1 year | A decrease in the amount of pelvic rotation after a single-event  multilevel surgery with FDRO. | The control group had preoperatively: normal foot progress angle and (almost) normal hip rotations. |
| Kwon 2013 | 25 | 28 | Single-event multilevel surgery with or without FDRO included | 1 year | Hip extension, rotation, and adduction improved significantly after surgery in the FDRO group. Foot progression angle improved in both groups, but the degree of improvement was significantly greater in the FDRO group. | The control group had preoperatively: normal foot progress angle and (almost) normal hip rotations. |
| Schwartz 2014 | 740 limbs | 348 limbs | Single-event multilevel surgery with or without FDRO included | 1 year | Limbs with anteversion and significant internal hip  rotation during gait benefit from an FDRO, but limbs with excessive anteversion and only, mild internal  Hips that are at risk of developing an excessive external foot progression angle. | Clusters were identified on a large retrospective cohort (1088 limbs): 1, had an FDRO, and had significant internal hip rotation gait preop 2, had an FDRO without significant preop. internal hip rotation gait  3, mixed cluster  4, no FDRO, no rotational malalignment |
| Thielen  2018 | 79 | 8 | Single-event multilevel surgery with or without FDRO included | 1 year | Supracondylar FDRO results in increased frontal hip moments. Changes in anteversion directly influence hip kinetics, although no direct change of the proximal bony geometry is performed. | The control group had preoperatively: normal foot progress angle and (almost) normal hip rotations. |
| Kay 2004 | 19 | 40 | Single-event multilevel surgery with or without FDRO included | 1 year | Improvement in pelvic rotation for bilaterally and unilaterally involved CP patients, both with and  without FDRO. | The control group had preoperatively: normal foot progress angle and (almost) normal hip rotations. |

**G. Supplementary figures:**

Fig.S1: PRISMA flowchart of study selection

**Identification of studies via databases and registers**

Records removed *before screening*:

Duplicate records removed
(n = 2016)

Records identified from:

Databases (n = 3443)

CINAHL: 276

Cochrane: 35
Embase: 955

PubMed: 608

Scopus: 873

Web of science: 696

**Identification**

Records excluded

(n = 1349)

Records screened

(n = 1427)

Cohen’s kappa 0.9

Reports not retrieved

(n = 3)

Reports sought for retrieval

(n = 78)

**Screening**

Cohen’s kappa 0.98

Reports excluded:

Conference lecture/poster (n=7)

No FDRO (n = 13)

No 3D gait analysis (n = 5)

Ineligible population (n=4)

Reports assessed for eligibility

(n = 75)

Studies included in review

(n = 26)

Reports of included studies

(n = 46)

**Included**

Fig.S2. Sensitivity analysis of hip rotation results. Data originating from high and low-quality articles did not differ. Therefore, all data were kept for statistical analysis.

Fig.S3. Sensitivity analysis of foot progression angle results. Data originating from high and low-quality articles did not differ. Therefore, all data were kept for statistical analysis.

Fig.S4a. Pooled Gait Deviation Index (GDI) results (mean change)

Fig.S4b. Pooled Gait Deviation Index (GDI) results (mean change) – leave on out analysis with GMFCS IV. patients left out

Fig.S5. Pooled Gait Profile Score (GPS) results (mean change in degrees)

Fig.S6. Pooled Gillette Gait Index (GGI) results (mean change)

Fig.S7. Pooled pelvic rotation results. Results are presented in degrees. Data was grouped post-hoc according to preoperative asymmetry. The asymmetrical group (more than 5 degrees of rotation preoperatively, 109 patients) had a significant improvement of 6.64 degrees. Heterogeneity was moderate. The symmetrical group (238 patients) had a minimal mean change of 1 degree. Heterogeneity was high. Test for subgroup difference confirms a statistically significant difference between the two groups.

Fig.S8 Pooled pelvic tilt results (mean change in degrees)

Fig.S9. Pooled knee flexion/extension (mean change in degrees)

Fig.S10. Pooled hip ab/adduction (mean change in degrees)

Fig.S11 Cadence ((mean change in steps/min)

Fig.S12. Step length (mean change in centimeters)

Fig.S13. Stride length (mean change in centimeters)

Fig.S14. Step width (mean change in centimeters) Step width reached statistical significance, most probably because the distance of the FOOT markers is assessed and not because there is a fundamental difference, meaning that the foot progress angle changed, not the step width.

Fig.S15. Velocity (mean change in meters/sec)

Fig.S16. Hip rotation subgroups distal vs. proximal FDRO localization (mean changes in degrees)

Fig.S17. Foot progression angle subgroups distal vs proximal FDRO localization (mean changes in degrees)

Fig.S18. Hip rotation FRDO vs. no-FDRO (mean differences in degrees)

Fig.S19. Foot progress FDRO vs no FDRO (mean differences in degrees)

**H: Adverse events**: El Barbary et al. described a percutaneous, distal FDRO technique. Five patients (9.8%) had light discharge from the Schanz screw entry point with superficial infection, all resolved within 10 days. Two patients (3.92%) developed mild knee flexion, which improved through stringent physiotherapy to 5° and 10° at 12-month follow-up. In one case (1.92%), a Schanz screw broke in the sixth postoperative week; no revision was needed.

Saglam et al. report infection in two patients (2.15%) in the distal FDRO group, which was cured with antibiotic therapy—non-union in two patients (2.15%), treated with revision platingand bone graft. Three fractures (3.23%) around the implants required surgical treatment.

Ounpuu (2000) et al. discuss that two patients (10%) in the distal FDRO group were noted to have angulation of their osteotomy into flexion (20° and 45°), with the latter requiring a revision of the osteotomy 6 weeks after surgery. This last patient was maintained for 5 months in a spica cast. There were no pathologic fractures or other surgical complications in the study group. No patient had a blood loss of >200 ml. Ounpuu (2017): One patient (5%) was treated with a distal FDRO, and K-wire fixation required revision at three weeks due to malunion into flexion. All osteotomies healed without additional complications.

Thompson reports 2 transient trochanteric bursitis, 3 transient neuropathic pain in the minimal-invasive group, and one failure of fixation requiring revision when the patient fell at home five weeks postop.

Kay, Kim, Ha Young, Moisan, Wren, and Piripris stated no complications or adverse events were observed.

**I: Definition of a good correction in the included articles:**

Aminian: hip rotation within the mean of typically developing children±1SD
Böhm: hip rotation within the mean of typically developing children±1SD

Boyer: hip rotation within the 10th to 90th percentile of typically developing children

Braatz 2013: less than 10° improvement in mean hip rotation in stance

Church: mean hip rotation during stance at Vshort >1 SD internal of the normal mean, and did not improve by 10 degrees compared with Vpre.

de Moralis: hip rotation within the mean of typically developing children±1SD

Desailly: An improvement of more than one standard deviation was classified as“improved.”

Elbarbay:’ Three of our patients were presented with under correction and persistent in-toeing with inward-directed patellae.’

Niklasch: postoperative mean hip rotation in the stance phase in a range of ±15° was defined as good results. (=Tipically developing±1.5 SD)

Ounpuu: mean hip rotation is within -5 to 10 (approx. range of typically developing children)

Vermuyten: good correction: mean hip rotation between -15° and 15

**J: Funnel plots:** Funnel plots and classical Egger’s test p-values for mean differences (MD) effect size were calculated. Study bias was considered low if the p-value was less than 10%. In case of fewer than 10 data sets, p-values can not be considered. Instead, a visual inspection of the plots should be carried out. Symmetrical alignment suggests a small probability of bias.

**1**. Funnel plot: gait scores

2 Funnel plot: pelvic rotation

**3**. Funnel plot: hip rotation

4. Funnel plot: Foot progression angle

5. Funnel plot: Pelvic tilt

6. Funnel plot: Knee flexion/extension (Although p value<0.05, we do not consider p values if less than 10 data sets were analyzed. The symmetry of the plot also suggests a low probability of publication bias)

7 Funnel plot: hip ab/adduction

8. Funnel plot: Cadence

9. Funnel plot: Step length

10. Funnel plot: stride length

11. Funnel plot: Step width

12. Funnel plot: Velocity

**J: articles excluded from the full-text phase and the explanation of the exclusion
*7 articles were abstracts of conference lectures or posters:*** Ounpuu 1989 ^8^, Schwartz 2013 ^9^, Church 2015 ^10^, Khouri 2017 ^11^, Langerak 2019 ^12^, Hueanerts 2020 ^13^, Royle 2020 ^14^

***In 13 articles surgery did not contain FDRO for all included patients:***

<50% had FDRO: Rutz 2013 ^15^, Aiona 2012 ^16^, Westwell 2009 ^17^, Lofterod 2008 ^18^, Zwick 2001 ^19^ Stefko 1998 ^20^;

50% had FDRO: Steele 2022 ^21^, Church 2022 ^22^, Chang 2004^23^;

65% had FDRO: Edwards 2021 ^24^, Dobson 2005 ^25^;

80% had FDRO: Min 2020 ^26^;

states multilevel surgery, but does not categorize it further: Schwartz 2016 ^27^

***In 5 articles instrumented 3D gait analysis lacked:***

Glessner 1969 ^28^, Cobeljic 2006 ^29^, Amichai 2009 ^30^, Skiak 2015 ^31^, Sarikaya 2020^32^

***In 4 articles population was not children with Cerebral Palsy:***

Lerch 2022 ^33^, Geisbüsch 2022 ^34^, MacWilliams 2016 ^35^, Dodgin 1998 ^36^

***We could not access the full text for the following 3 articles:***

Hoffer 1981 ^37^, Marherbe 1990 ^38^, Lawnicak 2010 ^39^

**K. References in the supplementary file:**

1. Cimolin V, Galli M. Summary measures for clinical gait analysis: a literature review. *Gait Posture*. Apr 2014;39(4):1005-10. doi:10.1016/j.gaitpost.2014.02.001

2. McMulkin ML, MacWilliams BA. Application of the Gillette Gait Index, Gait Deviation Index and Gait Profile Score to multiple clinical pediatric populations. Article. *Gait and Posture*. 2015;41(2):608-612. doi:10.1016/j.gaitpost.2015.01.005

3. Schwartz MH, Rozumalski A. The Gait Deviation Index: a new comprehensive index of gait pathology. *Gait Posture*. Oct 2008;28(3):351-7. doi:10.1016/j.gaitpost.2008.05.001

4. Baker R, McGinley JL, Schwartz MH, et al. The gait profile score and movement analysis profile. *Gait Posture*. Oct 2009;30(3):265-9. doi:10.1016/j.gaitpost.2009.05.020

5. Baker R, McGinley JL, Schwartz M, Thomason P, Rodda J, Graham HK. The minimal clinically important difference for the Gait Profile Score. *Gait Posture*. Apr 2012;35(4):612-5. doi:10.1016/j.gaitpost.2011.12.008

6. McMulkin ML, MacWilliams BA. Intersite variations of the Gillette Gait Index. *Gait Posture*. Oct 2008;28(3):483-7. doi:10.1016/j.gaitpost.2008.03.002

7. Schutte LM, Narayanan U, Stout JL, Selber P, Gage JR, Schwartz MH. An index for quantifying deviations from normal gait. *Gait Posture*. Feb 2000;11(1):25-31. doi:10.1016/s0966-6362(99)00047-8

8. Ounpuu S, Andrews M, Gage JR. Effects of internal tibial derotational osteotomies on gait in children with cerebral palsy. Article. 1989;22(10):1066. doi:10.1016/0021-9290(89)90410-7

9. Schwartz MH, Rozumalski A, Novacheck TF. Identifying historical selection criteria for femoral derotational osteotomy using the random forest algorithm. Conference Abstract. *Gait and Posture*. 2013;38:S41-S42. doi:10.1016/j.gaitpost.2013.07.083

10. Church. Persistence and recurrence following femoral derotational osteotomy in ambulatory children with cerebral palsy. Poster. *Developmental Medicine & Child Neurology*. 2015;57:48-48. doi:10.1111/dmcn.76_12887

11. Khouri N, Desailly E. Contribution of clinical gait analysis to single-event multi-level surgery in children with cerebral palsy. *ORTHOPAEDICS & TRAUMATOLOGY-SURGERY & RESEARCH*. FEB 2017;103(1):S105-S111. doi:10.1016/j.otsr.2016.11.004

12. Langerak NG, Tam N, du Toit J, Fieggen AG, Lamberts RP. Gait Pattern of Adults with Cerebral Palsy and Spastic Diplegia More Than 15 Years after Being Treated with an Interval Surgery Approach: Implications for Low-Resource Settings. Article. *Indian Journal of Orthopaedics*. 2019;53(5):655-661. doi:10.4103/ortho.IJOrtho_113_19

13. Huenaerts C, Desloovere K, Schreurs H, Van Campenhout A. Effect of simultaneous femoral derotation osteotomy and selective dorsal rhizotomy on gait in children with cerebral palsy: A pilot study. *Gait & Posture*. 2020;81:165-166. doi:10.1016/j.gaitpost.2020.07.117

14. Royle A, Pratt E, Milner L, Lecount L, Dickens W. A pre- and postoperative evaluation of femoral derotation osteotomy on coronal plane kinematics and kinetics of children with cerebral palsy. *Gait & Posture*. 2020;81:305-307. doi:10.1016/j.gaitpost.2020.08.049

15. Rutz E, Baker R, Tirosh O, Brunner R. Are Results After Single-event Multilevel Surgery in Cerebral Palsy Durable? *CLINICAL ORTHOPAEDICS AND RELATED RESEARCH*. MAR 2013;471(3):1028-1038. doi:10.1007/s11999-012-2766-9

16. Aiona M, Calligeros K, Pierce R. Coronal plane knee moments improve after correcting external tibial torsion in patients with cerebral palsy. Conference Paper. *Clinical Orthopaedics and Related Research*. 2012;470(5):1327-1333. doi:10.1007/s11999-011-2219-x

17. Westwell M, Ounpuu S, Deluca P. Effects of orthopedic intervention in adolescents and young adults with cerebral palsy. *Gait & Posture*. 2009;30(2):201-206. doi:10.1016/j.gaitpost.2009.04.012

18. Lofterød B, Terjesen T. Results of treatment when orthopaedic surgeons follow gait-analysis recommendations in children with CP. Article. *Developmental Medicine and Child Neurology*. 2008;50(7):503-509. doi:10.1111/j.1469-8749.2008.03018.x

19. Zwick EB, Saraph V Fau - Linhart WE, Linhart We Fau - Steinwender G, Steinwender G. Propulsive function during gait in diplegic children: evaluation after surgery. *J Pediatr Orthop B*. 2001;10(3):226-33.

20. Stefko RM, De Swart RJ, Dodgin DA, et al. Kinematic and kinetic analysis of distal derotational osteotomy of the leg in children with cerebral palsy. Article. *Journal of Pediatric Orthopaedics*. 1998;18(1):81-87. doi:10.1097/00004694-199801000-00016

21. Steele KM, Schwartz MH. Causal Effects of Motor Control on Gait Kinematics After Orthopedic Surgery in Cerebral Palsy: A Machine-Learning Approach. Article. *Frontiers in Human Neuroscience*. 2022;16doi:10.3389/fnhum.2022.846205

22. Church C, Biermann I, Lennon N, et al. Walking activity after multilevel orthopedic surgery in children with cerebral palsy. Article. *Developmental Medicine and Child Neurology*. 2022;64(10):1289-1296. doi:10.1111/dmcn.15228

23. Chang WN, Tsirikos AI, Miller F, Schuyler J, Glutting J. Impact of changing foot progression angle on foot pressure measurement in children with neuromuscular diseases. *GAIT & POSTURE*. AUG 2004;20(1):14-19. doi:10.1016/S0966-6362(03)00072-9

24. Edwards TA, Thompson N, Prescott RJ, Stebbins J, Wright JG, Theologis T. A comparison of conventional and minimally invasive multilevel surgery for children with diplegic cerebral palsy. Article. *Bone and Joint Journal*. 2021;103(1):192-197. doi:10.1302/0301-620X.103B1.BJJ-2020-0714.R1

25. Dobson F, Graham HK, Baker R, Morris ME. Multilevel orthopaedic surgery in group IV spastic hemiplegia. Article. *Journal of Bone and Joint Surgery - Series B*. 2005;87(4):548-555. doi:10.1302/0301-620X.87B4.15525

26. Min JJ, Kwon SS, Sung KH, Lee KM, Chung CY, Park MS. Factors affecting GDI improvement after single event multilevel surgery in patients with cerebral palsy. Article. *Gait and Posture*. 2020;80:101-105. doi:10.1016/j.gaitpost.2020.05.033

27. Schwartz MH, Rozumalski A, Steele KM. Dynamic motor control is associated with treatment outcomes for children with cerebral palsy. *DEVELOPMENTAL MEDICINE AND CHILD NEUROLOGY*. NOV 2016;58(11):1139-1145. doi:10.1111/dmcn.13126

28. Glessner JR, Jr. Derotation osteotomy of the femur in cerebral palsy. *Henry Ford Hosp Med J*. 1969;17(1):59-62 FAU - Glessner, J R Jr.

29. Cobeljić G, Djorić I Fau - Bajin Z, Bajin Z Fau - Despot B, Despot B. Femoral derotation osteotomy in cerebral palsy: precise determination by tables. *Clin Orthop Relat Res*. 2006;452:216-24.

30. Amichai T, Harries N Fau - Dvir Z, Dvir Z Fau - Patish H, Patish H Fau - Copeliovitch L, Copeliovitch L. The effects of femoral derotation osteotomy in children with cerebral palsy: an. *J Pediatr Orthop*. 2009;29(1):68-72 LID - 10.1097/BPO.0b013e3181924331 [doi].

31. Skiak E, Karakasli A, Basci O, Satoglu IS, Ertem F, Havitcioglu H. Distal femoral derotational osteotomy with external fixation for correction of excessive femoral anteversion in patients with cerebral palsy. *Journal of Pediatric Orthopaedics B*. 2015;24(5):425-432. doi:10.1097/BPB.0000000000000168

32. Sarikaya İA, Erdal OA, Şeker A, Görgün B, İnan M. Femoral derotation osteotomy in children with cerebral palsy using the pediatric proximal femoral nail. Article. *Journal of Pediatric Orthopaedics Part B*. 2020;29(1):15-21. doi:10.1097/BPB.0000000000000639

33. Lerch TD, Boschung A, Leibold C, et al. Less in-toeing after femoral derotation osteotomy in adult patients with increased femoral version and posterior hip impingement compared to patients with femoral retroversion. *JOURNAL OF HIP PRESERVATION SURGERY*. MAY 25 2022;9(1):35-43. doi:10.1093/jhps/hnac001

34. Geisbusch A, Gotze M, Putz C, Dickhaus H, Dreher T. Femoral derotation osteotomy-Does intraoperative electromagnetic tracking reflect the dynamic outcome? *JOURNAL OF ORTHOPAEDIC RESEARCH*. JUN 2022;40(6):1312-1320. doi:10.1002/jor.25168

35. MacWilliams BA, McMulkin ML, Davis RB, Westberry DE, Baird GO, Stevens PM. Biomechanical changes associated with femoral derotational osteotomy. *GAIT & POSTURE*. SEP 2016;49:202-206. doi:10.1016/j.gaitpost.2016.07.002

36. Dodgin DA, De Swart RJ, Stefko RM, Wenger DR, Ko JY. Distal tibial/fibular derotation osteotomy for collection of tibial torsion: Review of technique and results in 63 cases. *JOURNAL OF PEDIATRIC ORTHOPAEDICS*. JAN-FEB 1998;18(1):95-101. doi:10.1097/00004694-199801000-00018

37. Hoffer MM, Prietto C, Koffman M. Supracondylar derotational osteotomy of the femur for internal rotation of the thigh in the cerebral palsied child. Article. *Journal of Bone and Joint Surgery - Series A*. 1981;63(3):389-393. doi:10.2106/00004623-198163030-00011

38. Malherbe V, Lacert P. Femoral derotation osteotomy in cerebral palsy. Article. *Annales de Readaptation et de Medecine Physique*. 1990;33(3):249-260.

39. Ławniczak D, Jóźwiak M, Manikowska F. Assessment of absolute knee joint linear and angular velocity in patients with spastic cerebral palsy after operative treatment of lever arm disfunction deformities--prospective study. Article. *Chirurgia narzadów ruchu i ortopedia polska*. 2010;75(2):92-97.
